# Ubiquitin-Mediated Proteolysis as a Regulator of the Plant Defense-Growth Balance

**DOI:** 10.3390/plants15030506

**Published:** 2026-02-06

**Authors:** Matheus França Gonçalves, Aline Köhn Carneiro, Rodrigo de Miranda Otero, Adriana Silva Hemerly

**Affiliations:** Laboratório de Biologia Molecular de Plantas, Instituto de Bioquímica Médica, Universidade Federal do Rio de Janeiro, Cidade Universitária, Rio de Janeiro 21941-902, Brazil; mathheusfg@gmail.com (M.F.G.); aline.carneiro@bioqmed.ufrj.br (A.K.C.); rodrigooterobio@gmail.com (R.d.M.O.)

**Keywords:** ubiquitination, E3 ubiquitin-ligase, cell cycle progression, immune system, plant development

## Abstract

To survive in challenging environments, plants must rapidly activate immune responses while maintaining developmental plasticity and reproductive success. This requires continuous negotiation of limited energy and metabolic resources between growth, development, and defense. Ubiquitin-mediated proteolysis has emerged as a versatile regulatory mechanism that may integrate immune responses with plant developmental programs. In this review, we summarize accumulating evidence that ubiquitination shapes immune responses at multiple regulatory levels. Many of these immune-regulatory mechanisms depend on ubiquitin-dependent pathways that also govern developmental processes and cell cycle regulation. This overlap points to shared molecular nodes that integrate defense with growth. This functional overlap provides a mechanistic basis for growth–defense trade-offs and highlights how plants optimize fitness under stress conditions. Together, these findings position ubiquitin-mediated proteolysis as a unifying regulatory framework through which plants integrate immune responses with developmental programs and cell cycle control. This coordination helps maintain resilience and productivity in a fluctuating environment.

## 1. Introduction

To survive in challenging environments, plants must rapidly activate immune responses while preserving developmental plasticity and reproductive success. This balance depends on regulatory systems capable of integrating environmental cues with endogenous developmental programs [1].

Biotechnology-assisted breeding has greatly advanced the development of pathogen-resistant cultivars [2]. However, resistance is often accompanied by developmental trade-offs [3], since many resistance sources rely on constitutive activation or increased basal activity of endogenous or exogenous genes. This sustained defense redirects substantial energy and metabolic resources away from growth processes to defense mechanisms. As a consequence, plants often exhibit reduced biomass and stature, as well as nutritional imbalances that generally lead to yield penalty [1,4]. A classic example is the barley *Mlo* gene [5]. Early studies showed that recessive alleles generated by loss-of-function mutations (*mlo*) confer broad-spectrum resistance to *Erysiphe graminis* f. sp. *hordei* (now classified as *Blumeria hordei*), while also inducing a leaf lesion phenotype associated with cell death [5]. It was later found that this locus lies within a chromosomal region containing QTLs linked to early maturity and reduced grain yield [6]. Recently, pyramiding *Pi* genes (*Pib*, *Pi25*, and *Pi54*) in rice were shown to greatly enhance resistance to *Magnaporthe oryzae*, although these lines may show a potential reduction in productivity [7].

Despite these drawbacks, yield losses may be acceptable when the target disease is widespread and causes severe damage in susceptible cultivars. In these scenarios, the physiological cost of resistance is offset by its agronomic benefits [8]. This was demonstrated in wheat, where, under high disease severity, resistant cultivars experienced proportionally smaller yield losses than susceptible ones, highlighting the agronomic advantage of resistance under adverse conditions [9]. In general, the costs associated with resistance can be inferred to act directly or indirectly on cell cycle regulation. This is because plant growth fundamentally depends on the orderly progression of this process. Consequently, any reduction in growth typically reflects some degree of repression of cell cycle activity [10].

Ubiquitination has emerged as one versatile mechanism potentially capable of integrating immune responses with plant developmental programs. Initially characterized primarily as a signal for protein degradation, ubiquitination is now recognized as a multifunctional regulatory code that governs protein stability, localization, activity, and interaction networks. Through the coordinated action of ubiquitin-conjugating enzymes, ligases, and deubiquitinases, the ubiquitin–proteasome system (UPS) enables rapid and reversible control of key signaling hubs. These hubs shape stress adaptation, immunity, cell cycle and growth [11].

In this review, we first summarize the current knowledge on the Ubiquitin Proteasome System (UPS) in plants, followed by its independent roles in modulating the plant cell cycle and plant immunity. We then discuss available evidence suggesting that plant UPS might integrate some immune responses with plant developmental programs. We emphasize mechanisms involved in balancing effective defense with plant growth.

## 2. The Ubiquitin Proteasome System (UPS) in Plants

Ubiquitination is one of the most abundant and versatile post-translational modifications [12]. Initially associated mainly with protein turnover and cellular homeostasis, it is now recognized as a key regulator of diverse cellular processes. This includes signal transduction, intracellular trafficking, cell cycle checkpoint control, and the coordination of cellular responses to biotic and abiotic stimuli [13,14,15].

### 2.1. The Ubiquitination Machinery

The plant resilience is tightly linked to cellular responses elicited by environmental and endogenous cues. The maintenance of protein homeostasis depends on regulatory mechanisms that control protein abundance, spatial distribution, post-translational modification, and turnover [16,17]. Within this regulatory framework, the covalent conjugation of ubiquitin (Ub) to target proteins functions as a central molecular signal that determines diverse protein fates [18].

**Figure 1:**
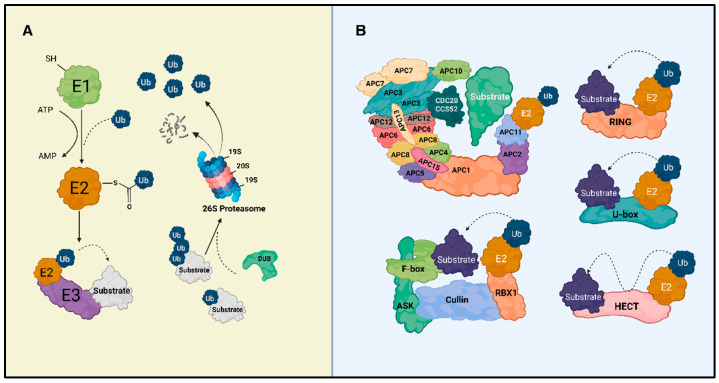
The Ubiquitin–proteasome system in plants. (**A**) Representation of the ubiquitination pathway dependent on the cascade of enzymes E1, E2, and E3, culminating in substrate ubiquitination and subsequent degradation by the 26S proteasome complex. E1 activates ubiquitin in an ATP-dependent manner, which is then transferred to the conjugating enzyme E2. The E3 ligase recognizes the specific substrate and facilitates the transfer of ubiquitin from E2 to the substrate. DUBs (deubiquitinases) remove ubiquitin molecules for recycling. (**B**) Main classes of E3 ligases in plants. The Anaphase-Promoting Complex/Cyclosome (APC/C) recognizes substrates through the coactivators CDC20 and CCS52A/B. Below, the SCF (SKP1–Cullin–F-box) complex is formed by the core subunits ASK, CULLIN, and RBX1, together with the F-box protein that confers substrate specificity. On the right, the main catalytic domains of E3s (RING, U-box, and HECT) are shown, which differ in their mechanisms of ubiquitin transfer to the substrate. Solid arrows indicate the direction of biochemical events; dashed arrows indicate ubiquitin movement. Created in BioRender. Köhn carneiro, A. (2026) https://BioRender.com/j7nw3sq (accessed on 1 February 2026).

Ubiquitin conjugation is mediated by a conserved enzymatic cascade consisting of three sequential steps. First, the ubiquitin-activating enzyme (E1) catalyzes ATP-dependent adenylation of the Ub C-terminal glycine, followed by the formation of a thioester bond with its active-site cysteine. Activated Ub is then transferred to the catalytic cysteine of a ubiquitin-conjugating enzyme (E2) [19]. In the final step, E2 associates with a ubiquitin ligase (E3), which confers substrate specificity and facilitates the covalent attachment of Ub to lysine residues within the target protein [20]. This reaction can result in mono-ubiquitin attachment or the assembly of polyubiquitin chains via any of the seven internal lysine residues of Ub (Lys6, Lys11, Lys27, Lys29, Lys33, Lys48, and Lys63). These modifications generate structurally and functionally distinct signals [19,21] (Figure 1A).

These Ub-based modifications constitute the molecular entry point for pathways directing substrates toward specific cellular outcomes. Canonical Lys48-linked chains serve as the primary signal for recognition by the 26S proteasome, leading to proteolytic degradation. In contrast, Lys63-linked polymers are predominantly associated with non-proteolytic functions, including endocytosis, intracellular trafficking, and signal transduction [22]. Beyond protein turnover, Ub conjugation modulates diverse regulatory processes such as DNA repair, transcriptional control, and stress-responsive signaling networks. This is particularly important in plant adaptive responses to environmental challenges and in plant immunity [23]. Accordingly, this system enables rapid and reversible control of protein function in both developmental and stress-induced contexts [22].

Prior to or during proteasomal processing, Ub chains are removed or remodeled by deubiquitinating enzymes (DUBs). These enzymes hydrolyze isopeptide bonds and allow Ub molecules to be recycled, thereby maintaining the cellular Ub pool and ensuring the efficiency of this regulatory system [24] (Figure 1A).

Among the three enzyme classes involved in the ubiquitination cascade, E3 ligases constitute the largest and most extensively characterized group. These enzymes play a pivotal role in the ubiquitin–proteasome system (UPS). They mediate the interaction between E2 conjugating enzymes and target substrates, thereby acting as the primary determinants of pathway specificity [25]. E3 ligases can be identified based on their catalytic mechanisms or on substrate-recruitment domains through sequence analysis approaches [25]. Such strategies have led to the identification of approximately 1400 E3 ligases in *Arabidopsis thaliana* [26]. There are three major structural families of E3 ligases: HECT, RING, and U-box. Table 1 and Figure 1B summarize the different types of E3 ligases currently described in plants.

**Figure 2:**
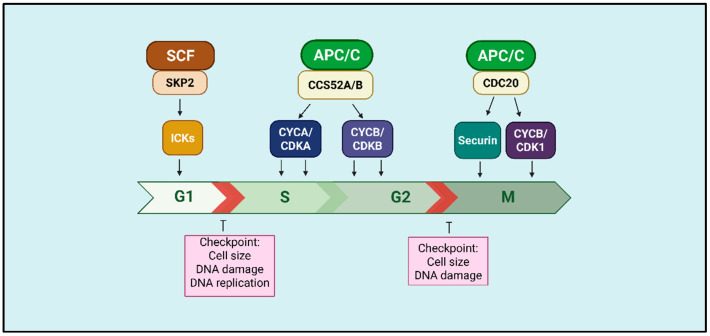
Role of SCF and APC/C complexes in plant cell cycle progression. SCF^SKP2^ regulates the G1/S transition through the degradation of CDK inhibitors (ICKs), while APC/C^CCS52A/B^ acts during the S/G2 transition and APC/C^CDC20^ promotes progression from metaphase to anaphase by degrading securin and mitotic cyclins. Checkpoints ensure genomic integrity and control of cell size at each phase. Created in BioRender. Köhn carneiro, A. (2026) https://BioRender.com/auu151v (accessed on 1 February 2026).

Two E3 ubiquitin ligase complexes play central and complementary roles in the precise control of cell cycle phase transitions: the anaphase-promoting complex/cyclosome (APC/C) and the Skp1–Cullin–F-box (SCF) complex [49] (Table 1 and Figure 2). These checkpoint mechanisms tightly coordinate cell cycle progression between phases, ensuring both genome integrity and the irreversibility of cell cycle events. The APC/C drives cell cycle progression by targeting key mitotic regulators, including mitotic cyclins (CYCA and CYCB) and securins, for ubiquitination and subsequent proteasomal degradation. Conversely, the SCF complex facilitates the timely activation of CDK–cyclin complexes by promoting the degradation of CDK inhibitors, thereby enabling entry into S phase [14] (Figure 2).

The same principles that govern ubiquitin-dependent proteolysis in growth-related processes also support plant defense responses. This view positions ubiquitination as a central regulatory module at the interface between development and immunity.

### 2.2. New Frontiers in Ubiquitination Research

Research on plant ubiquitination has advanced substantially over the past decade [58]. Early studies focused on individual components of the ubiquitin–proteasome system and their direct substrates, often linking specific ubiquitination events to developmental or stress-related phenotypes. Although essential for establishing the biological relevance of this pathway, these approaches provided a limited view of ubiquitin-mediated regulation [59]. Advances in high-throughput technologies and integrative omics strategies have since enabled system-level analyses. These studies reveal ubiquitination as a dynamic and context-dependent regulatory layer that integrates plant development, immunity, and environmental adaptation [60].

A major methodological advance has been the development of ubiquitinomics, a proteomics-based approach that employs diverse purification strategies, such as the enrichment of di-glycine-modified peptides, to enable large-scale mapping of ubiquitination sites across the plant proteome [61]. These studies have demonstrated that ubiquitination is a widespread modification affecting proteins involved in a broad range of cellular functions. In a study conducted in *Arabidopsis thaliana*, 17,940 ubiquitinated lysine sites were identified from 6453 proteins across multiple tissues. Furthermore, gene ontology analysis of these ubiquitinated proteins revealed their involvement in a wide variety of biological processes [62]. This approach has also been applied to economically important crop species, such as rice. In a study using rice panicles, 1638 ubiquitinated lysine sites were identified across 916 unique proteins. In addition, this work revealed three conserved ubiquitination motifs, in which glutamic acid and aspartic acid residues were most frequently found near to ubiquitinated lysines, suggesting conserved sequence features associated with ubiquitin attachment [63].

Ubiquitinomics has emerged as a powerful approach to dissect the dynamics of plant physiological responses to stress, particularly during biotic interactions and immune activation. In maize (*Zea mays*) infected with maize chlorotic mottle virus (MCMV) and sugarcane mosaic virus (SCMV), viral infection was associated with a global increase in protein ubiquitination relative to non-infected plants [64]. Lysine-ubiquitinated proteins were predominantly linked to photosynthesis and central carbon metabolism, including fructose and mannose metabolism as well as glyoxylate and dicarboxylate pathways, indicating extensive metabolic reprogramming during viral challenge [64].

Converging evidence further indicates that ubiquitination acts in concert with other post-translational modifications rather than in isolation. This principle is illustrated by an integrated proteomic analysis in rose (*Rosa* sp.) infected with *Botrytis cinerea*, in which coordinated changes in protein phosphorylation and ubiquitination were observed [65]. Cross-layer comparison revealed a subset of proteins dynamically regulated by both modifications, many of which correspond to putative pattern-triggered immunity components at the plasma membrane. These findings underscore the importance of post-translational modification crosstalk in fine-tuning immune signaling and reinforce ubiquitination as a central node in multilayered regulatory networks [65].

At the same time, increasing attention has focused on the degronome, defined as the complete set of degradation signals embedded within the proteome [66,67]. Degrons consist of sequence elements or structural motifs that are recognized by specific E3 ubiquitin ligases, thereby conferring selectivity to ubiquitin-dependent protein turnover [68]. The integration of computational degron prediction approaches, including machine learning–based models trained on curated reference datasets, has been critically important for the development of degron recognition tools and for advancing the systematic identification of protein degradation targets [69,70].

## 3. Ubiquitination as a Positive or Negative Modulator of the Plant Immune System

Beyond its classical role in protein degradation, accumulating evidence demonstrates that ubiquitination plays critical regulatory functions in plant metabolism and immunity [13,71,72,73]. E3 ubiquitin ligases, the most extensively characterized components of this pathway, can act as either positive or negative regulators of immune responses, depending on their substrates. They also participate in stress perception, signal transduction, hormone signaling, transcriptional reprogramming, and programmed cell death [74].

The activation of defense mechanisms relies on complex networks of post-translational modifications, with ubiquitination emerging as a central regulatory hub that shapes receptor stability, signaling amplitude, and the duration of defense responses. Ultimately, this coordination regulates downstream outputs such as Ca^2+^ influx, reactive oxygen species (ROS) production, stomatal closure, and the biosynthesis of defense-related phytohormones [75].

**Figure 3:**
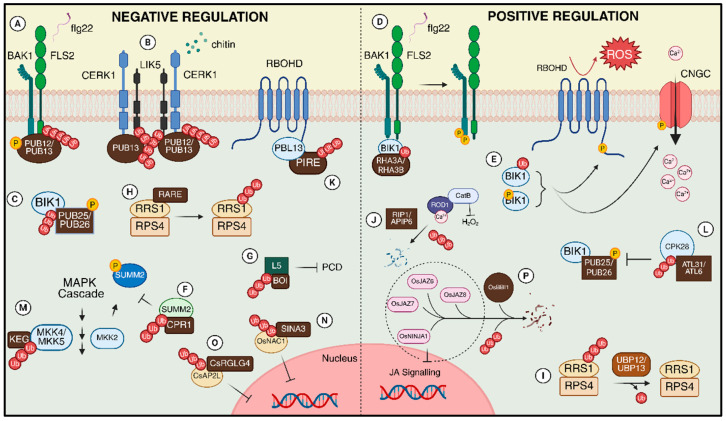
Examples of positive and negative regulation of plant immunity mediated by ubiquitination. (**A**) Negative regulation of FLS2; (**B**) Negative regulation of CERK1 and LIK5; (**C**) Negative regulation of BIK1; (**D**) Positive regulation of BIK1 and the FLS2-BAK1 complex; (**E**) Monoubiquitinated BIK1 positively regulates RBOHD and CNGC; (**F**) Negative regulation of SUMM2; (**G**) Negative regulation of L5 and PCD; (**H**) Negative regulation of the RRS1/RPS4 immune receptor pair via ubiquitination of the WRKY domain; (**I**) Positive regulation of RRS1/RPS4 by UBP12/UBP13 deubiquitinase activity; (**J**) Positive regulation of CatB through the destruction of ROD1; (**K**) Negative regulation of RBOHD; (**L**) Positive regulation of BIK1 through the destruction of CPK28; (**M**) Negative regulation of the MAPK cascade; (**N**) Negative regulation of the OsNAC1 transcription factor; (**O**) Negative regulation of the CsAP2L transcription factor; (**P**) Positive regulation of jasmonic acid signaling. Further details on the role of each component are provided in the text. The Arrows indicate regulatory outcomes of the indicated molecular events. Created in BioRender. Köhn carneiro, A. (2026) https://BioRender.com/0g10m2g (accessed on 1 February 2026).

In Figure 3, we present examples that will be discussed in detail in the following sections, illustrating how different types of ubiquitin ligases target distinct substrates and can positively or negatively modulate the plant immune system.

### 3.1. Ubiquitination and Pathogen-Associated Stress Perception

Flagellin, a major component of bacterial flagella, induces the recruitment of two U-box type E3 ubiquitin ligases, PLANT U-BOX 12 (PUB12) and PLANT U-BOX 13 (PUB13), to the FLS2 (FLAGELLIN SENSING 2) receptor complex (LRR-RLK) in Arabidopsis [44]. Upon flagellin perception, FLS2 associates with BRASSINOSTEROID INSENSITIVE1 (BRI1) ASSOCIATED KINASE (BAK1) (another LRR-RLK), which phosphorylates PUB12 and PUB13. This phosphorylation facilitates the formation of the FLS2-PUB12/13 complex, promoting polyubiquitination and degradation of FLS2 (Figure 3A). Furthermore, *pub12* and *pub13* mutants exhibited enhanced immune responses upon flagellin treatment, indicating that PUB12/13 act as negative regulators of FLS2-mediated signaling through direct ubiquitination of the receptor [44]. Subsequent studies investigated other types of pattern-recognition receptors (PRRs), such as the chitin receptor complex containing the lysin-motif (LysM) receptor-like kinases LYSIN MOTIF-CONTAINING RECEPTOR-LIKE KINASES 5 (LYK5) and CHITIN ELICITOR RECEPTOR KINASE 1 (CERK1). Upon fungal chitin perception, CERK1 phosphorylates the receptor-like cytoplasmic kinase PBS1-LIKE PROTEIN 27 (PBL27), which subsequently activates the intracellular mitogen-activated protein kinase (MAPK) cascade. PUB12 and PUB13 were shown to interact with the intracellular domain of CERK1. Like the previous findings, the Arabidopsis *pub12/pub13* mutant displayed enhanced chitin-induced immune responses, including increased ROS production, MAPK activation, and callose deposition. These results suggest that PUB12 and PUB13 also function as negative regulators of the chitin receptor complex (Figure 3B) [76]. Similar results were seen in a study with the LYK5 receptor [45] (Figure 3B). PRRs and their co-receptors, such as BAK1 and CERK1, interact with intracellular molecules to activate downstream immune signaling. Among these molecules are receptor-like cytoplasmic kinases (RLCKs), such as BIK1. It has been shown that the U-box type E3 ligases PLANT U-BOX 25 (PUB25) and PLANT U-BOX 26 (PUB26) mediate the ubiquitination of BIK1, targeting it for proteasomal degradation (Figure 3C). This mechanism highlights another layer of negative regulation of immunity through the ubiquitination of receptor-associated components [46].

Conversely, BIK1 monoubiquitination has been described as a positive regulatory mechanism of immunity. The Ring type E3 ligases RHA3A (RING-H2 FINGER A3A) and RHA3B catalyze BIK1 monoubiquitination, enabling its dissociation from the FLS2–BAK1 complex, as well as BAK1-mediated phosphorylation of BIK1 (Figure 3D). This process is essential for the activation of immune signaling. Once dissociated, BIK1 regulates the production of ROS through the phosphorylation of plasma membrane-localized NADPH oxidases and triggers cytosolic calcium influx via the phosphorylation of cyclic nucleotide-gated channels (CNGCs) (Figure 3E) [32].

Like PRRs, ubiquitination has also been linked in literature to regulatory processes involving NLRs. The activation of the NLR SUPPRESSOR OF MKK1 MKK2, 2 (SUMM2) is mediated by the MAPK kinase kinase MEKK2, leading to autoimmunity. However, the F-box E3 ligase CONSTITUTIVE EXPRESSER of GENE1 (CPR1) regulates SUMM2 abundance through ubiquitination and subsequent proteasomal degradation, thereby functioning as a negative regulator of immunity (Figure 3F) [77]. Likewise, the RING-type ubiquitin ligase BOTRYTIS SUSCEPTIBLE 1 INTERACTOR (BOI) has been associated with the ubiquitination of the CC-NBS-LRR protein L5, modulating its stability (Figure 3G). L5 has been reported as an inducer of programmed cell death in *Nicotiana benthamiana* [78]. In Arabidopsis, the RESISTANCE TO RALSTONIA SOLANACEARUM 1 and RESISTANCE TO PSEUDOMONAS SYRINGAE 4 (RRS1/RPS4) immune receptor pair mediates disease resistance by detecting pathogen effectors via the integrated WRKY domain of RRS1. The balance of the RRS1/RPS4 receptor complex is finely regulated by ubiquitination and deubiquitination. The RING E3 ligase RARE binds directly to the WRKY domain of RRS1, leading to its proteasomal degradation and weakening the complex’s activity (Figure 3H). In contrast, the deubiquitinases UBP12 and UBP13 also interact with the WRKY domain of RRS1, removing ubiquitin chains and thereby restoring its stability and function in plant immunity (Figure 3I) [33].

In addition to their already established roles in regulating protein stability, studies suggest the involvement of Ubiquitin and UPS related proteins in other processes in eukaryotes. For instance, they can influence liquid-liquid phase separation (LLPS) and the formation of biomolecular condensates [79]. In this context, the ubiquitination state, including the actions of deubiquitinases, can modulate the biophysical properties of target proteins and their interaction networks [80].

Although there is still no direct evidence of LLP regulated by plant deubiquitinases such as UBP12 or UBP13, analogous mechanisms in other eukaryotic models suggest that reversible deubiquitination can influence the dynamic partitioning of immune receptor complex into condensates [81]. Since polyubiquitin chains allow greater accessibility to ubiquitin-blind surfaces, proteins such as UBIQUILIN-2 (UBQLN2) can promote LLPS, thereby enabling a transition between homotypic and partially heterotypic LLPS [79].

### 3.2. Ubiquitination in Signal Transduction

Upon pathogen recognition, immune receptors trigger a cascade of signal transduction events that rapidly reprogram cellular activities. Early responses include ion fluxes, particularly calcium influx, which activates calcium-dependent protein kinases (CDPKs) and mitogen-activated protein kinase (MAPK) cascades. These kinases, in turn, phosphorylate various downstream targets, including transcription factors, leading to broad transcriptional reprogramming. In parallel, ROS are also produced, and together with other signals, they help amplify the immune response and activate defense mechanisms [82].

Ubiquitination plays distinct roles in different branches of the signal transduction cascade. In rice (*Oryza sativa*), for instance, the *ROD1* (*RESISTANCE OF RICE TO DISEASES 1*) gene encodes a C2 domain Ca^2+^ sensor that functions as a global regulator of immunity. Loss of function of this gene has been linked to enhanced resistance against a broad spectrum of pathogens, since the sensor can activate catalase and promote the degradation of H_2_O_2_, a key molecule in triggering defense responses. It has been shown that the ubiquitin RING-type ligases RIP1 (ROD1 INTERACTING PROTEIN 1) and APIP6 (AvrPiz-t INTERACTING PROTEIN 6) regulate the stability of ROD1, thereby playing an essential role in the positive regulation of plant immunity (Figure 3J) [83].

The production of ROS during pathogen attack is mediated by members of the NADPH oxidase family, known in plants as respiratory burst oxidase homologs (RBOHs), which generate O_2_^−^ in the apoplast. Among them, RBOHD is a membrane-localized protein and the major family member in Arabidopsis. Following pathogen-associated molecular pattern (PAMP) perception, a rapid Ca^2+^ influx induces conformational changes in RBOHD and its phosphorylation by CDPKs, leading to ROS production. It has been shown that the RLCK PBS1-like 13 (PBL13) associates with phosphorylates conserved residues in the C-terminal region of RBOHD, a process critical for its stability and activity. Furthermore, the ubiquitin E3 RING-type ligase PLANT IMMUNITY RING-type E3 ligase (PIRE) directly interacts with both PBS-LIKE PROTEIN 13 (PBL13) and the C-terminal of RBOHD, marking it for degradation and providing an additional regulatory layer that controls RBOHD accumulation and activity (Figure 3K) [35].

CALCIUM-DEPENDENT PROTEIN KINASE 28 (CPK28) plays a central role in immune regulation, as it functions within the BIK1 immune hub. As previously noted, PUB25/26 polyubiquitinate BIK1, targeting it for degradation [84]. The ligase activity of PUB25 is enhanced when it is phosphorylated at Thr95 by CPK28. Moreover, CPK28 itself is regulated through ubiquitination and proteasomal degradation mediated by the E3 ligases ARABIDOPSIS TOXICOS EN LEVADURA 31 (ATL31) and ATL6, whose activity is induced by the elicitor flg22. As a result, ATL31 and ATL6 contribute positively to BIK1 stability and the activation of immune responses (Figure 3L) [84].

The mitogen-activated protein kinase (MAPK) signaling cascade is one of the most critical pathways associated with plant immune responses. In Arabidopsis, the protein levels of MKK4 and MKK5 are regulated by the ubiquitin ligase KEEP ON GOING (KEG) (Figure 3M). Moreover, the protein kinase ENHANCED DISEASE RESISTANCE1 (EDR1) also modulates the levels of MKK4 and MKK5 and negatively affects KEG phosphorylation. The phosphorylation sites identified in KEG appear to be essential for its accumulation. This study uncovered a complex mechanism by which plants fine-tune immune responses: on the one hand, by regulating MAPK cascade members through ubiquitination; and on the other, by modulating the ubiquitination cascade itself via phosphorylation. These two interconnected processes are crucial for the precise regulation of plant defense [34].

Ultimately, the signal transduction cascade triggered during biotic stress converges on the regulation of gene expression. Over the years, numerous studies have demonstrated the central role of ubiquitination in controlling various transcription factors. In rice, for instance, the activity of the transcription factor WRKY45—a key regulator of the salicylic acid/benzothiadiazole-induced defense program—is modulated by the nuclear ubiquitin–proteasome system. In this study, inhibition of the 26S proteasome by MG132 led to the accumulation of polyubiquitinated WRKY45 and a transient increase in the expression of WRKY45 target genes. These findings suggest that WRKY45 undergoes continuous degradation via the UPS to prevent unwarranted activation of the defense system in the absence of infection signals [85].

In tomato (*Solanum lycopersicum*), the transcription factor DOMAIN-CONTAINING PROTEIN 1 (NAC1), a member of the NAC family (NAM, ATAF1/2, CUC2), is involved in regulating plant defenses and its abundance is controlled by the RING type ubiquitin ligase SEVEN IN ABSENTIA 3 (SINA3) (Figure 3N). During *Pseudomonas* infection, *SINA3* is downregulated, whereas *NAC1* is upregulated, indicating that SINA3 functions as a negative regulator of the NAC1-dependent immune response [36].

More recently, in citrus (*Citrus sinensis*), it was shown that overexpression of the RING type ubiquitin ligase RING-type Glyceraldehyde-3-phosphate dehydrogenase–Like Gene 4 (CsRGLG4) significantly enhanced resistance to green mold (*Penicillium digitatum*). The authors demonstrated that CsRGLG4 directly interacts with the transcription factor APETALA2-LIKE (CsAP2L), regulating its abundance. These findings indicate that CsRGLG4 modulates citrus fruit resistance to *P. digitatum* by fine-tuning CsAP2L activity via ubiquitination (Figure 3O) [37].

### 3.3. Ubiquitination in Hormone Signaling

Plant defense responses are characterized by a profound transcriptional reprogramming that affects multiple metabolic pathways, among which hormonal signaling plays a central role. Increasing evidence indicates that this hormone-dependent reprogramming is closely linked to ubiquitination and the subsequent proteasomal degradation of key regulators [86].

Recent studies show that ubiquitination of fine-tunes hormone signaling not only by regulating protein stability, but also by modulating receptor activity and subcellular localization. Specifically, the RING-type E3 ubiquitin ligase RING FINGER OF SEED LONGEVITY 1 and the CULLIN4–DAMAGE-SPECIFIC DNA-BINDING PROTEIN 1 (CUL4–DDB1) WD protein–type E3 ubiquitin ligase complex promote polyubiquitination and degradation of abscisic acid (ABA) receptors known as Pyrabactin Resistance 1 (PYR1)/PYR1-like (PYL)/Regulatory Components of ABA Receptors (RCARs). In contrast, DOA10A-mediated monoubiquitination enhances ABA receptor localization to the plasma membrane and improves signal perception [87].

It was also demonstrated that auxin regulates plant growth not only through canonical SCF^TIR1/AFB^-mediated degradation of Aux/IAA repressors, but also via a redox-based mechanism in which auxin-induced nitric oxide (NO) promotes S-nitrosylation of key UPS components, including E3 ligase subunits [88]. This modifies their activity, stability and interactions and integrates hormonal and redox signaling to fine-tune proteasome-dependent control of auxin responses and downstream developmental programs in plants.

In Arabidopsis, WAVY GROWTH RING-type E3 ubiquitin ligases regulate the polar localization of PIN-FORMED auxin efflux carriers by limiting their missorting into basal trafficking pathways, independently of PIN phosphorylation. This mechanism shapes directional auxin transport and developmental patterning, underscoring a proteasome-independent role of ubiquitination in hormone signaling [89].

In a study on wheat infected with Chinese wheat mosaic virus (CWMV), ubiquitome analysis, performed using a combination of affinity enrichment and high-resolution liquid chromatography coupled to tandem mass spectrometry. This is revealed through bioinformatic analysis that one of the most significantly enriched pathways was hormonal signaling, along with metabolic processes, stress response, plant–pathogen interaction, and ribosomal pathways [90]. Similarly, in *Nicotiana benthamiana* leaves infected with Tomato brown rugose fruit virus (ToBRFV), combined ubiquitome and proteome profiling identified differentially ubiquitinated proteins. Enrichment analysis indicated that ToBRFV infection increased the ubiquitination levels of proteins associated with ion transport, MAPK signaling pathways, and plant hormone signal transduction [91]. Together, these studies underscore the significant role of ubiquitination in regulating hormonal signaling during the plant antiviral response.

In Arabidopsis, the transcriptional activators linked to salicylic acid (SA) and ethylene, NONEXPRESSOR OF PATHOGENESES-RELATED GENES 1 (NPR1) and ETHYLENE-INSENSITIVE 3 (EIN3), are not solely regulated by their specific ligases (such as CRL3 for NPR1 or SCF^EBF^ for EIN3). They also undergo a “relay” process involving HECT-type ligases associated with the proteasome, UBIQUITIN PROTEIN LIGASE 3 (UPL3) and UBIQUITIN PROTEIN LIGASE 4 (UPL4). These ligases finalize the degradation of the activator proteins, ensuring that hormone-modulated immune responses occur with tightly controlled duration and intensity [28].

In tomato, the RING type ubiquitin ligase ARABIDOPSIS TOXICOS LETHAL2-LIKE (SlATL2) is induced both upon infection with *Pseudomonas syringae* pv. tomato (Pst) DC3000 and following treatment with the defense phytohormones salicylic acid (SA) and jasmonic acid (JA). SlATL2 functions as a negative regulator of the immune response by limiting reactive oxygen species production and callose deposition, targeting the COP9 signalosome subunit 5a (SlCSN5a), a positive regulator of tomato immunity. Proteasomal degradation of SlCSN5a suppresses the expression of SA-dependent defense genes. Thus, SlATL2 plays a key role as a negative modulator of tomato immune responses to flg22 perception [38].

In rice, the RING-type E3 ubiquitin ligase BLAST AND BTH-INDUCED1 (OsBBI1) plays a central role in modulating jasmonic acid (JA) signaling. It mediates the ubiquitination and degradation of multiple components of the JA signaling pathway, including proteins that act as repressors of the immune response, thereby conferring broad-spectrum resistance to multiple races of the blast fungus. By regulating the abundance of proteins such as JASMONATE ZIM-DOMAINS PROTEIN 6, 7 and 8 (OsJAZ6, OsJAZ7 and OsJAZ8), and NOVEL INTERACTOR of JAZ 1 (OsNINJA1), OsBBI1 fine-tunes both the intensity and duration of the JA-mediated immune response, ensuring effective defense against pathogens (Figure 3P) [39].

### 3.4. Ubiquitination in Programmed Cell Death

The plant immune response can also trigger programmed cell death (PCD). Unlike necrosis—which results from irreversible and disorganized cellular damage—PCD is characterized by coordinated molecular and biochemical events that lead to the controlled self-destruction of the cell. A typical manifestation of this process is the hypersensitive response (HR), which locally restricts infection by inducing the rapid death of cells adjacent to the invasion site. HR is often associated with the production of ROS, modifications in ion fluxes, particularly Ca^2+^ influx, and the activation of kinase cascades such as MAPKs and CDPKs [92,93].

Ubiquitination has also been shown to play a crucial role in regulating programmed cell death (PCD). In rice, the U-box/ARM ubiquitin ligase SPOTTED LEAF 11 (SPL11) and its Arabidopsis ortholog PUB13 act as negative regulators of PCD. In this context, SPL11 was found to interact with SPIN6 (SPL11-interacting protein 6), targeting it for ubiquitination and subsequent degradation via the 26S proteasome pathway. Rice plants with SPIN6 silencing through RNAi, as well as mutant lines, exhibited enhanced pathogen resistance, which was accompanied by the activation of defense-related genes and increased ROS production [47].

Lesion Mimic Mutants (LMMs) in rice constitute a class of mutants capable of spontaneously forming necrotic lesions in plant tissues. These lesions resemble the hypersensitive response (HR) but occur in the absence of pathogen infection. Multiple studies have shown that most rice LMMs confer durable and broad-spectrum disease resistance. Many genes within the LMM family are associated with ubiquitination [94]. For example, OsCUL3a/SPL88, a component of a RING-type ubiquitin ligase, interacts with NPR1, promoting its degradation; the LM phenotype of the cul3a mutant results from NPR1 accumulation [40]. Another recent example is REGULATORY PARTICLE TRIPLE-A ATPase 5A (OsRPT5A), a regulatory 6A subunit of rice proteasome. A point mutation in the eighth exon leads to the LM phenotype, and functional complementation as well as CRISPR/Cas9-generated knockouts confirmed OsRPT5A’s role in controlling this trait. These findings suggest that OsRPT5A plays a key role in regulating ROS homeostasis and enhancing pathogen resistance in rice [95].

Deubiquitinases have also been linked to plant immunity. In rice, both loss-of-function and overexpression of the LMM22 gene triggered a hypersensitive response (HR)-like phenotype, accompanied by reactive ROS accumulation and activation of defense responses. LMM22 encodes a ubiquitin-specific protease (UBP) that interacts with SPL35, a protein containing a CUE domain (coupling of ubiquitin conjugation to ER degradation), identified as a novel component in the regulation of cell death and immune responses in plants. This study suggests that LMM22 positively regulates SPOTTED LEAF 35 (SPL35) abundance through its deubiquitination activity [96].

Programmed cell death (PCD) can also occur through the process of autophagy. The formation of the autophagosome involves two pathways analogous to the ubiquitin system. In the AUTOPHAGY-RELATED PROTEIN 12 (ATG12)-mediated pathway, for instance, ATG12 is activated by the enzyme ATG7 (analogous to an E1). Subsequently, the ATG10 gene encodes an E2-like conjugating enzyme, which is essential for facilitating the covalent linkage between ATG12 and ATG5 [97]. In plants, autophagy has been shown to target virulence factors for degradation. For example, the βC1 factor of Cotton leaf curl Multan virus (CLCuMuV) is directed for degradation through its interaction with ATG8 [98]. Interestingly, earlier studies reported that βC1 proteins from geminiviruses can subvert the host ubiquitination machinery to assist their helper viruses in infecting plants [99].

## 4. Potential Involvement of Ubiquitin-Mediated Proteolysis in Modulating the Plant Growth/Defense Balance

As discussed in the previous sessions, the independent roles of ubiquitin-mediated proteolysis as a positive or negative modulator of the cell cycle machinery and the plant immune system are well established. This highlights ubiquitination as a versatile mechanism potentially capable of coordinating the growth/defense balance in plants. Therefore, an important question is whether UPS components are shared in the modulation of both processes.

**Figure 4:**
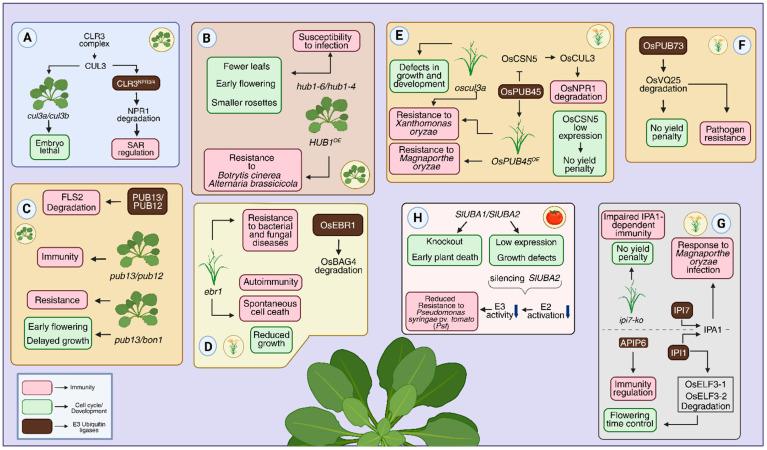
Developmental consequences of UPS modulation in plants. (**A**) The CUL3-based E3 ligase complex regulates degradation of NPR1. (**B**) HUB1, a RING-type E3 ligase, enhances resistance to pathogens such as *Botrytis cinerea*. (**C**) U-box-type E3 ligases PUB12 and PUB13 regulate FLS2 receptor turnover through degradation, attenuating immune responses. (**D**) The OsBAG4 gene, regulated by OsEBR1. (**E**) OsCULLIN3 plays roles in immunity and development. (**F**) OsPUB73, another E3 ligase, promotes degradation of OsVQ25, conferring pathogen resistance. (**G**) IPA1, a transcription factor that regulates IPI1 by controlling the OsELF3-1/2 proteins, modulates flowering time and immune responses through APIP6. (**H**) The ubiquitin-activating enzyme SlUBA1/2 is required for immunity. The Arrows indicate regulatory outcomes of the indicated molecular events. Created in BioRender. Köhn carneiro, A. (2026) https://BioRender.com/0g10m2g (accessed on 1 February 2026).

In the following subsection, we first highlight aspects related to plant developmental processes, and in the next, we compile molecular evidence on how these components may influence cell-cycle regulation. They are summarized in Table 2, and some examples are highlighted in Figure 4.

### 4.1. Crosstalk of UPS Between Plant Immunity and Plant Development

Some evidence of ubiquitin-mediated proteolysis regulating plant immunity as well as plant development were described in the model plant Arabidopsis, and in agronomically important plants.

#### 4.1.1. Arabidopsis

The CRL3 complex is an E3 ubiquitin-ligase composed of the scaffold protein CULLIN3 (CUL3), which anchors the other components: RBX1 (RING-box protein 1) and the BTB/POZ (Broad-Complex, Tramtrack, Bric-a-brac) adaptor protein [50]. In plant immunity, the main complex described is CRL3^NPR3/4^, which targets NPR1 for degradation during defense responses, thereby regulating systemic acquired resistance (SAR) [51]. In parallel, previous work demonstrated that the two CUL3 isoforms, CUL3A and CUL3B, are essential for embryonic development. The double mutant *cul3a/cul3b* is embryo lethal (Figure 4A) [100].

The loss-of-function mutants for the RING E3 ligase gene *HISTONE MONOUBIQUITINATION1* (*HUB1*), *hub1-6* and *hub1-4* alleles, showed high susceptibility to infection by necrotrophic fungi *Botrytis cinerea* and *Alternaria brassicicola*, whereas *HUB1* overexpression conferred resistance to these pathogens [98] (Figure 4B). In addition, the *hub1-6* and *hub1-4* mutants displayed noticeably smaller rosettes with fewer leaves, and early flowering, compared with the wild-type (Col-0) [41,101]. The genes *MADS AFFECTING FLOWERING 1* (*MAF1*) and *MAF4*, members of the *FLOWERING LOCUS C* (*FLC*) gene family and known repressors of flowering, were strongly repressed in the loss-of-function mutants. In contrast, *FLOWERING LOCUS T* (*FT*) was highly induced, consistent with the observed early-flowering phenotype (Figure 4B) [41].

PUB13, along with its partner PUB12, has been described as a negative regulator of immunity by targeting the flagellin-induced receptor FLS2 for degradation [44] and the *pub12* and *pub13* mutants showed enhanced immune responses upon flagellin treatment [44] (Figure 4C). PUB13 also regulates flowering time in a photoperiod-dependent manner, as the *pub13* loss-of-function mutant exhibits early flowering under middle-day or long-day conditions, but not under short-day conditions [102,103]. Recently, it was demonstrated that PUB13 interacts with the copine protein BONZAI 1 (BON1), which is a direct substrate of PUB13. Knockout of *BON1* in the *pub13* mutant enhanced the delayed growth and early flowering phenotypes and further increased the mutant resistance to different biotrophic pathogens (Figure 4C) [104].

Ectopic overexpression of PLANT U-BOX 41 (OsPUB41) in Arabidopsis enhanced callose deposition in the cell wall and increased the expression of jasmonic acid-related and infection-responsive genes. As a result, *OsPUB41^OE^* lines exhibited increased tolerance to *Rhizoctonia solani* AG1-IA, supporting a positive role for PUB41 in innate immunity [105]. Consistently, AtPUB41 is broadly expressed in vegetative and reproductive tissues, and the *pub41* mutant shows reduced seed dormancy, decreased sensitivity to ABA during germination, and increased drought sensitivity, indicating that AtPUB41 also positively regulates ABA signaling [106].

PUB2 and PUB4 positively regulate pattern-triggered immunity by promoting efficient signal transduction downstream of pattern recognition receptors. Loss-of-function *pub2* and *pub4* mutants display compromised PTI responses, including reduced ROS burst, attenuated MAPK activation, and enhanced susceptibility to *Pseudomonas syringae*. PLANT U-BOX PROTEIN 2 AND 4 (PUB2 and PUB4) are associated with key immune signaling components, such as FLS2, BIK1 and RbohD, and facilitate the assembly and stabilization of immune signaling complexes. Despite their established role in immunity, *pub2* and *pub4* mutants also exhibit reduced rosette growth. This suggests that these E3 ligases contribute to vegetative development and supports the idea that UPS components coordinating immune signaling often exert parallel functions in plant growth regulation [107].

The UBIQUITIN PROTEIN LIGASE (UPL) family consists of seven members harboring a C-terminal HECT domain. Among them, UPL1, UPL3, and UPL5 function as positive regulators of salicylic acid-mediated gene expression and enhance plant immunity [29]. In contrast, the *upl4* null mutant displayed the opposite phenotype, showing normal resistance to *Psm* ES4326 under low inoculum levels. In parallel, the *upl3/upl4* double mutant exhibited reduced growth, early senescence, and low seed production compared with the respective single mutants [29].

CPR1 is an SCF F-box protein known to promote the degradation of NLR-type R proteins, such as SNC1 and RPS2, acting as a negative regulator of plant immunity [108]. This regulation is essential to prevent constitutive activation of immunity in the absence of pathogen pressure and to maintain cellular homeostasis. Loss-of-function mutants of *CPR1* resulted in defense-associated dwarfism [108]. Recently, a novel E3 ligase involved in *R* gene homeostasis, termed MUSE16 (MODULATOR OF SNC1-MEDIATED IMMUNITY 16), was described. Unlike *cpr1*, the *muse16* mutant does not exhibit defense-associated dwarfism. Instead, they accumulate RPS2, suggesting that this protein may be a direct degradation target of MUSE16. This might account for the absence of a defense-related dwarf phenotype in *muse16* [109].

#### 4.1.2. Agronomically Important Plants

OsEBR1 (ENHANCED BLIGHT AND BLAST RESISTANCE 1) is a RING-type E3 ubiquitin ligase in rice (*Oryza sativa*) described as balancing strong disease resistance with normal plant growth (Figure 4D) [42]. OsEBR1 targets OsBAG4, a member of the BAG (Bcl-2–associated athanogene) protein family, for ubiquitination-mediated degradation to maintain innate immune homeostasis. The *ebr1* mutant showed increased resistance to a wide range of bacterial and fungal pathogens, but this enhanced defense was accompanied by spontaneous programmed cell death, autoimmunity, and reduced growth [42].

OsCULLIN3 (OsCUL3) is an E3 ligases (CRL3) in rice that also plays a role in both plant immunity and growth regulation [52]. *oscul3a* mutant plants showed enhanced resistance to infection by the pathogen *Xanthomonas oryzae* pv. *oryzae*. However, this increased resistance was accompanied by defects in growth and development, including suppressed panicle growth and reduced elongation of the first internode. Furthermore, grains from these mutants exhibited greater accumulation of total lipids accumulation at the expense of starch and protein, which were reduced in the mutant grains. The mutant plants also showed alterations in specific pathways of fatty acid metabolism, including linoleic acid and alpha-linolenic acid. These pathways are activated by the increased presence of specific enzymes called Lipoxygenases (LOXs), specifically CHLOROPLAST MEMBRANE-ASSOCIATED LIPOXYGENASE 1 (CM-LOX1/2), generating lipid peroxides (oxidized lipids), which can trigger cell death and plants immune response. Meanwhile, the leaves showed elevated H_2_O_2_ levels [52].

Recently, OsCUL3 was shown to be stabilized by OsCSN5 (COP9 SIGNALOSOME SUBUNIT 5), which promotes the degradation of OsNPR1, a positive regulator of immunity, thus preventing its constitutive activation in the absence of pathogens [110]. In contrast, OsCSN5 itself is ubiquitinated by the E3 ligase OsPUB45. Overexpression of *OsPUB45* enhanced rice resistance to *Magnaporthe oryzae* and *Xanthomonas oryzae* pv. *oryzae*, whereas its dysfunction reduced resistance. Rice plants with reduced *OsCSN5* expression via RNAi did not show any yield penalties in the evaluated parameters, including plant height, grain length, grain width, effective panicle number per plant, grain number per panicle, seed setting rate, and thousand-grain weight (Figure 4E) [110].

OsPUB73 is an E3 ligase that was demonstrated to positively regulate rice resistance to a broad spectrum of pathogens through the degradation of the OsVQ25 protein [103]. *osvq25* null mutants showed enhanced pathogen resistance and did not display any growth penalties (Figure 4F) [111].

The transcription factor IPA1 (Ideal Plant Architecture 1) plays a central role in promoting both immunity and productivity in rice plants through phosphorylation of the serine 163 residue and the coactivator IPI7 (IPA1 interactor 7), an E3 ligase that polyubiquitinates IPA1 at lysine 29 [112]. IP7 is required for the activation of the expression of immune-related genes in response to infection by *Magnaporthe oryzae*. Rice Nipponbare plants lacking IPI7 showed impaired IPA1-dependent immunity but did not display any yield penalties as shown by the panicle size, the number of primary and secondary panicle branches, and the total number of grains per panicle [112].

Another E3 ligase that interacts with IPA1, termed IPI1, is a regulator of both immunity and flowering in rice [113]. IPI1 controls flowering time and immune responses by directly ubiquitinating to EARLY FLOWERING3-1/2 (OsELF3-1 and OsELF3-2), targeting them for degradation in an E3 ligase activity-dependent manner. In addition, IPI1 regulates rice immunity by stabilizing the E3 ligase APIP6 in a manner independent of its own E3 ligase activity. Loss of *IPI1* function promoted early flowering and compromised immune responses in rice plants [113]. In an antagonistic manner, *IPI1* overexpression led to delayed flowering, a phenotype similar to that observed in *oself3-1* mutants (Figure 4G) [114].

In cotton, the U-box ubiquitin ligase MOSA4-ASSOCISTE COMPLEX PROTEIN3e (GhMAC3e) is ubiquitously expressed across plant tissues and is induced by infection with *Verticillium dahliae*, as well as by other stimuli [115]. Silencing of *GhMAC3e* in cotton delayed primary stem growth and reduced plant biomass, accompanied by decreased auxin levels in petioles and leaf veins. In contrast, ectopic overexpression of *GhMAC3e* in Arabidopsis significantly enhanced cotton resistance to Verticillium wilt [115].

The pathogen *Xanthomonas vasicola* pv. *musacearum* (Xvm), which causes banana *Xanthomonas* wilt in East Africa, is of major economic importance to the region. Some edited knockout line of the *MusaPUB22/23* genes in banana exhibited complete resistance to the pathogen, whereas others showed partial resistance compared with wild-type plants. Notably, no significant growth or yield penalties were observed in these lines [116].

Although much is known about ubiquitin ligases in plant immunity, far less is understood about other components of the ubiquitin–proteasome system (UPS). Recently, two tomato E1 ubiquitin-activating enzymes, SlUBA1 and SlUBA2, were shown to differentially regulate plant development and immunity (Figure 4H) [117]. Reduced expression of either gene caused defects in growth and development, while complete silencing resulted in severe abnormalities that led to plant death between five and seven weeks of age. In terms of immune responses, only S1UBA2 silencing compromised resistance to *Pseudomonas syringae* pv. *tomato* (*Pst*) (Figure 4H) [117].

### 4.2. Crosstalk Between UPS Components in Cell Cycle and Immunity

Alterations in plant growth and development ultimately depend on changes in cell proliferation dynamics, which are tightly regulated by the cell cycle machinery. Although many studies describing immune-related phenotypes do not directly assess cell cycle regulators, the observed effects on organ growth, biomass accumulation, or developmental timing strongly suggest underlying modulation of cell cycle activity. In this context, the ubiquitin–proteasome system (UPS) emerges not merely as a shared component acting independently in growth and immunity, but as a regulatory platform capable of coordinating these processes. By controlling the stability and activity of key cell cycle regulators and immune signaling components, the UPS enables context-dependent adjustments that balance defense activation with sustained growth and development.

To illustrate the multifaceted nature of these interactions, Table 2 summarizes representative UPS components that participate simultaneously in cell cycle regulation and plant immunity. This comparative overview highlights how shared molecular players bridge cellular proliferation with stress adaptation mechanisms.

#### 4.2.1. CDC48: A Central ATPase Linking Proteostasis, Immunity, and Programmed Cell Death in Plants

The *CDC48* (*CELL DIVISION CYCLE 48*) gene was named because it was identified as part of the yeast (*Saccharomyces cerevisiae*) cell cycle mutant collection, in which mutants exhibited arrest primarily at the G2/M transition [118]. Subsequent studies revealed that *CDC48* encodes an AAA+ ATPase (ATPases Associated with diverse cellular Activities), highly conserved across eukaryotes [119]. This enzyme is a key component of the ubiquitin-proteasome system and is involved in multiple cellular processes, including membrane-associated protein degradation, DNA repair, gene expression, membrane fusion, and autophagy [120]. In the cell cycle, CDC48 has been shown to participate in late mitotic events by facilitating spindle disassembly at the end of mitosis, a process required for efficient mitotic exit [121].

In Arabidopsis, *AtCDC48* has been shown to act as a negative regulator of NLR-mediated immunity. Partial loss-of-function mutants of *atcdc48* displayed dwarf phenotypes and enhanced resistance to the oomycete *Hyaloperonospora arabidopsidis* Noco2, along with elevated levels of the NLR SNC1, highlighting its role in SNC1 protein turnover [122]. Later studies confirmed that CDC48 unfolds substrates after polyubiquitination for degradation by the 26S proteasome [123,124].

**Table 2:** Proteins potentially participating in the coordinated regulation of the cell cycle and plant immunity.

Protein Name	Complex Affiliation	Known Target/Function in Cell Cycle/Development	Known Target/Function in Immunity	Observed Growth-Defense Trade Off	References
CDC20-3 and CCS52B-2	APC/C activator subunits.	Activation and substrate recognition of APC/C driving cell cycle progression.	Silencing in *Triticum aestivum* induction enhances resistance to Chinese wheat mosaic virus (CWMV) infection.	Yes, enhanced viral resistance is associated with altered growth.	[56]
APC/C^CDC20^	APC/C E3 ligase complex.	Regulates cell cycle transitions, during the metaphase/anaphase transition and mitotic exit, via degradation of CYCA/B and securin.	Geminiviruses and criniviruses manipulate APC/C^CDC20^ to modulate RBR1 in tomato.	Yes, increased viral spread correlated with growth repression and endoreduplication.	[125]
OSD1 and UVI4	APC/C inhibitors.	Negative regulators of APC/C activity, restraining cell-cycle progression.	Overexpressing OSD1 and UVI4, indirect activation of *R* genes, such as *SNC1*, enhancing the immune response.	Yes, enhanced resistance is accompanied by dwarfism and altered leaf morphology in Arabidopsis.	[126]
CPR5	APC/C associated regulator.	Modulation of CYC levels (*CYCB1;1*, *CYCB1;2*, and *CYCB1;4*), affecting CDK–cyclin complex homeostasis.	Negative regulator of immune signaling against *Pseudomonas syringae*.	Yes, increased resistance is associated with smaller rosette size and reduced plant growth in Arabidopsis.	[127]
APC7-CT	APC/C structural subunit.	Derived from the APC7 subunit, generating stability of the APC/C complex.	APC7-CT shares a high homology with the tobacco IVR. Overexpression in Arabidopsis reduces susceptibility to both RNA and DNA viruses.	No, enhanced resistance without detectable growth penalty.	[128,129]
ACIF1	Core SCF complex components.	SCF complex mediated degradation of ICKs, inhibitors of CYC/CDK, regulate the transition between the G1 and S phases.	Formation of immune related SCF complex. Overexpression positively regulates resistance to *Verticillium dahliae* in Arabidopsis and cotton.	Yes, resistance is linked to altered growth and yellowing symptoms in *Arabidopsis* and cotton.	[130]
SCF^SKIP14^	SCF E3 ligase complex.	No direct role related.	Overexpressing to promote apple tree resistance against the fungal pathogen *Valsa mali* and reactive oxygen species accumulation.	Yes, enhanced defense is associated with reduced lesion expansion.	[131]
NpPP2-B10	SCF associated adaptor.	No direct role related.	Overexpression in tobacco promotes disease resistance by participating in the plant immune response via ubiquitin-proteasome pathway.	Yes, altered growth rate and seed germination rate, plant height accompany resistance.	[132]
CDC48	AAA-ATPase associated.	Protein extraction and remodeling during development.	Increase in CDC48 mobility in the plant cell nucleus leads to the renewal of resistance proteins, such as SNC1, limiting autoimmunity.	Yes, increased activity is linked to hypersensitive response and PCD.	[133]
PUB13/SPL11	U-box E3 ligase.	Indirect control of growth via regulation of programmed cell death.	It regulates plant defense responses dependent activation via SA-dependent pathway.	Yes, enhanced resistance is associated with reduced rosette size.	[91,103,134]
HUB1	RING E3 ligase.	Epigenetic regulation of growth-related gene expression.	Overexpression conferred resistance to fungi *Botrytis cinerea* and *Alternaria brassicicola*.	Yes, resistance coupled to early flowering and reduced rosette size in Arabidopsis.	[41]
PUB12/PUB13	U-box E3 ligases.	No direct role related.	Regulate FLS2 receptor turnover. Through degradation, attenuating immune responses.	Yes, enhanced resistance to early flowering, and delayed growth in Arabidopsis.	[104]
UPL family (UPL1-5)	HECT E3 ligases.	Transcription factor turnover affecting development.	Positive regulators of salicylic acid-mediated gene expression and enhance plant immunity.	Yes, reduced growth, decreased seed set and early senescence with enhanced defense in Arabidopsis.	[29]
OsBAG4	BAG-domain protein, UPS associated.	Regulation of growth and activation of cell death.	Enhanced resistance to bacterial and fungal pathogens.	Yes, resistance is associated with growth reduction and cell death activation in *Oryza sativa*.	[42]
OsPUB45/ OsCSN5/ OsCUL3	CUL3 based E3 ligase pathway.	Developmental regulation via NPR1 turnover.	Resistance to *X. oryzae* and *M. oryzae*.	Diverse, some genotypes show resistance without yield penalty in *Oryza sativa*.	[110]
OsPUB73/ OsVQ25	U-box E3 ligase.	No direct role related.	Degradation of the OsVQ25 protein enhancing resistance to pathogens.	No, resistance without detectable growth defects in *Oryza sativa*.	[111]
IPA1/IPI7	IPA1 regulatory complex.	Control of flowering time and developmental timing.	Required for IPA1-dependent immunity, induced resistance during *Magnaporthe oryzae* infection.	Yes, impaired immunity without strong growth defects in mutant in *Oryza sativa*.	[112]
IPA1/IPI1	Ubiquitin-mediated transcriptional regulator.	Regulation of flowering via OsELF31/2.	Stabilization of APIP6 to promote immune signaling.	Yes, early flowering is associated with reduced immunity in *Oryza sativa*.	[113]
GhMAC3e	U-box E3 ligase, UPS associated.	Regulation of stem growth.	Induced resistance by infection with *Verticillium dahlia*.	Yes, enhanced resistance is accompanied by altered growth in cotton.	[115]
SlUBA1/2	Ubiquitin-activating E1 enzymes.	Essential for normal plant development.	*SlUBA2* required resistance to *Pseudomonas syringae*.	Yes, immune defects are associated with severe developmental defects in tomato.	[117]

Interestingly, CDC48 itself is regulated by ubiquitination. A SNIPER (snc1-influencing plant E3 ligase reverse genetic) screen demonstrated that CDC48 interacts with SNIPER7, an F-box protein whose overexpression triggers autoimmunity. Overexpression of *SNIPER7* phenocopies the atcdc48 mutant, with reduced CDC48A levels, suggesting that CDC48 is a substrate of SCF^SNIPER7^-mediated ubiquitination [135].

Moreover, CDC48 plays a role in programmed cell death. Experiments with the elicitor cryptogein, produced by the oomycete *Phytophthora cryptogea*, showed that tobacco cell lines stably overexpressing *NtCDC48A1* exhibited early cell death [136]. In tobacco leaves treated with cryptogein, a hypersensitive response was observed at the infection site, confirming CDC48’s involvement in programmed cell death [137]. Recently, using Fluorescence Correlation Spectroscopy (FCS), it was observed that treatment with cryptogein leads to increased nuclear mobility of CDC48, as well as rapid interaction with numerous partners potentially involved in defense mechanisms [133].

#### 4.2.2. SCF-Mediated Coordination of Growth and Immune Responses

The SCF complex selectively ubiquitinates proteins, thereby linking processes such as hormone signaling, cell-cycle progression, and immunity through 26S proteasome-mediated degradation [138].

The activation of immune signaling pathways can alter the homeostasis of SCF complexes containing F-box proteins such as S-PHASE KINASE-ASSOCIATED PROTEIN2-A/B (SKP2A and SKP2B). Some pathogens can compete for components of the ubiquitin system, including ASK1 and CUL1 proteins [30] (Table 1). For example, *Ralstonia solanacearum* encodes type III secretion system (T3SS) that contains the F-box domain and has been shown to interact with the *Arabidopsis* SKP1 [139]. *Agrobacterium tumefaciens* F-box effector protein VirF has been shown to interact with plant homologs of the yeast Skp1 protein through the F-box domain [140].

**Figure 5:**
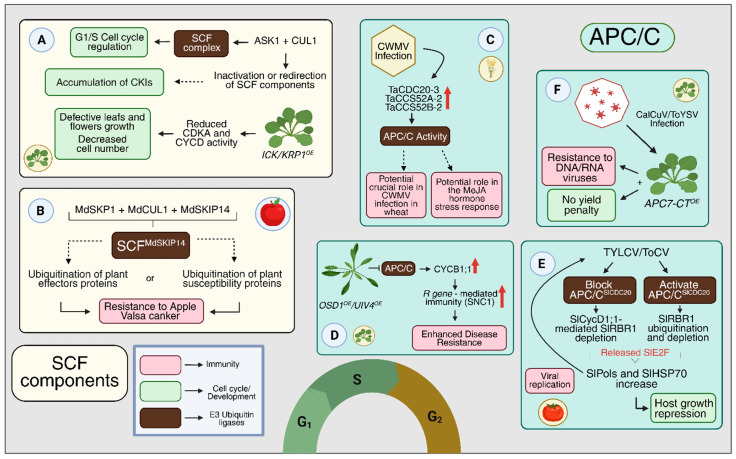
Integration of cell cycle regulation and immunity by ubiquitin-mediated pathways in plants. (**A**) Immune activation generates competition for the components (ASK1 and CUL1) leading to inactivation of the SCF^SKP2A/B^ complex and accumulation of ICK/KRP1, which reduces CDKA/CYCD activity. (**B**) The SCF^SKIP14^ complex (Md*SKP1*, Md*CUL1*, and Md*SKIP14*), promoting immune responses and resistance to Infection by *Valsa mali*. (**C**) CWMV infection in wheat increases the expression of APC/C regulators (Ta*CDC20-3* and Ta*CCS52*). (**D**) Inhibition of APC/C by OSD1 or UVI4 leads to CYCB1;1 accumulation, activating *R* gene-mediated immunity, such as *SNC1* and increasing resistance to *Pseudomonas syringae*. (**E**) TYLCV and ToCV viruses sequester APC/C in tomato plants, resulting in reduced growth. (**F**) Overexpression of *APC7-CT* confers resistance to DNA and RNA viruses without reducing plant productivity. The functional roles of each component are described in detail in the text and Table 2. The black arrows indicate regulatory outcomes or downstream consequences of the indicated molecular events. Created in BioRender. Köhn carneiro, A. (2026) https://BioRender.com/yqsb4x1 (accessed on 1 February 2026).

As a consequence of pathogen-mediated recruitment, sequestration, or inactivation of SCF components, the formation and activity of protein complexes involved in cell cycle progression may be impaired. This disruption can lead to the accumulation of cyclin-dependent kinase inhibitors (CKIs), thereby negatively affecting cell division. Studies in Arabidopsis have shown that overexpression of ICK/KRP1 reduces CDKA and CYCD activity, resulting in a decreased cell number and impaired growth of both vegetative and reproductive organs, producing leaves and flowers with a serrated phenotype (Figure 5A) [141]. This phenotype is also presented by some plants affected by pathogens (Table 1).

In addition, SCF components have recently been identified as directly involved in plant defense. MdSkp1 and MdCUL1 interact with the F-box protein MdSKIP14 to form the SCFSKIP14 complex, which acts as a positive regulator of resistance to Apple Valsa canker (AVC) caused by the fungus *Valsa mali* (Figure 5B) [131]. Together, these data corroborate the idea that immune activation and even pathogens can compete directly with development-associated SCF complexes, thus disrupting the balance between cell cycle progression and defense responses.

#### 4.2.3. Functional Versatility of CRL3 Complexes in Plant Development, Stress Adaptation, and Defense

One of the most studied BTB proteins (BROAD COMPLEX, TRAMTRACK, BRIC-À-BRAC) that form the CRL3 complex is the MATH domain-containing BTB protein (BPM) [53,142,143,144,145]. Studies have shown that two of the targets for the CRL3^BPM^ complex are MYB domain protein 56/106 (MYB56 and MYB106), members of one of the largest families of transcription factors in plants, and that it is a negative regulator of flowering time [53,146]. The CRL3^LRB^ complex has also been described as acting on flowering during the vernalization period by inducing the degradation of the FRIGIDA (FRI) protein [54].

Dehydration-responsive element binding 2A (DREB2A) [142] is also described as a target for the CRL3^BPM^ complex, where in the absence of BPM, the stabilization of DREB2A allows for greater tolerance to thermal stress. The interaction between the CRL3BPM complex also has as target the protein phosphatase 2C protein (PP2CA) [145] and the class I homeobox-leucine zipper (HD-ZIP) transcription factor ATHB6 [143]. The proteolysis of these targets acts in modifying signaling and response to ABA. More recently, a new BTB protein has been described as part of the CRL3 complex, the BTB/POZ protein hypersensitive to ABA 1 (BPH1), which negatively regulates ABA-mediated actions [147]. In relation to hormonal signaling, there is also the case of the ETO1 protein which acts as an adapter for the degradation of the 1-AMINOCYCLOPROPANE-1-CARBOXYLATE SYNTHASE 5 (ACS5) protein, thus regulating ethylene biosynthesis [148].

The CLR3^NPR3/4^ complex is also regulated by proteolysis and is mediated by the UBP12/UBP13 deubiquitinating proteins, which ensure the stability of the complex [51]. Other BTB proteins with proteolytic action in plant immunity have also been described. Among them are PUB22/23-INTERACTING PROTEIN 1 (POB1), which is responsible for degrading the PUB29 protein in apples, suppressing defense against *Botryosphaeria dothidea* [149]. In tobacco, POB1 acts by regulating the PUB17 protein and suppressing programmed cell death [150]. In soybeans, there is the GmBTB/POZ protein, which leads to the degradation of the GmAP2 factor, increasing resistance against *Phytophthora sojae* [150].

When searching for CRL3 complexes in plant cell cycle regulation, there is still no evidence of a direct link as occur with APC/C and CLR1 [57]. The main protein of a cell cycle regulating pathway, ARMADILLO BTB ARABIDOPSIS PROTEIN 1 (ABAP1), which acts during the licensing from the G1 to S phases [151], contains a BTB/Arm domain and has been shown to interact with CUL3 [152]. However, proteolytic activity has not yet been indicated. In this case, there is a need to discover more interactions between BTB proteins and targets to try elucidating other processes that they may be regulating or connecting.

#### 4.2.4. Emerging Functions of the APC/C Complex in the Regulation of Plant Defense

In wheat, the proteins CELL DIVISION CYCLE 20 (CDC20) and CELL CYCLE SWITCH 52 A/B (CCS52A/B), may also play roles in immunity. The expression levels of TaCCS52A-2, TaCCS52B-2, and TaCDC20-3 increased significantly after CWMV infection, and silencing through VIGS resulted in infection inhibition (Figure 5C) [56]. In addition, APC/C dysregulation can affect gene expression, including disease resistance (*R*) and plant immunity genes in Arabidopsis. Overexpression of *OMISSION OF SECOND DIVISION1* (*OSD1*) and *UV-INSENSITIVE 4* (*UVI4*), negative regulators of the APC/C complex, leads to enhanced plant immunity associated with *R* genes (Figure 5D) [126]. Although the connections are not yet fully understood, these findings suggest that certain viruses may exploit APC/C subunits to increase their virulence (Table 1).

A recent study demonstrated in tomato (*Solanum lycopersicum*) that both RNA and DNA viruses can co-opt the APC/C^CDC20^ complex to enhance their spread. APC/C^CDC20^ functions in the cell cycle by binding to RETINOBLASTOMA-RELATED 1 (RBR1) or mediating the ubiquitination of CYCD1, thereby maintaining the RBR1–E2F complex. Geminiviruses and Criniviruses have the ability to hijack the APC/C^CDC20^ complex, promoting RBR1 degradation through ubiquitination or inhibiting APC/C^CDC20^ activity to stimulate CYCD1-mediated RBR1 depletion (Figure 5E) [125].

In addition to its interactions with activating subunits and co-opted active complexes, the role of an APC/C subunit in the plant immune system has also been identified. Sequence analysis revealed that the C-terminal region of APC7 (APC7-CT) in Arabidopsis shares high homology with a tobacco viral replication inhibitor (IVR-like), which reduces plant susceptibility [129]. Moreover, Arabidopsis plants overexpressing *APC7-CT* exhibited lower susceptibility to both RNA and DNA viruses, along with improved growth (Figure 5F) [128].

## 5. Concluding Remarks and Perspectives

Growth–defense trade-offs are not simply the result of parallel and independent regulatory pathways but instead might reflect coordinated decisions at shared molecular nodes. Because plant growth fundamentally depends on cell proliferation, changes in developmental output often mirror alterations in cell cycle activity. Thus, regulatory systems that can fine-tune cell cycle progression emerge as critical integrators of immune activation and growth control. The ubiquitin–proteasome system (UPS), with its ability to rapidly and reversibly modulate the abundance and activity of key regulatory proteins, is particularly well suited for this integrative function. In this review, we present evidence that the UPS, by targeting key components of immune signaling pathways as well as core cell cycle regulators, might provide a mechanistic framework through which plants coordinate defense activation with developmental plasticity, rather than simply prioritizing one process over the other. To further investigate this hypothesis, studies are necessary to biochemically and genetically unravel how the mechanisms operate.

Another key question to be addressed is how the UPS regulation of growth-defense balance is also connected with abiotic environmental signals. UPS is a central regulator of protein homeostasis in eukaryotic organisms. In plants, whose sessile lifestyle imposes unique adaptive constraints, this regulatory layer must operate with exceptional precision to ensure survival in fluctuating and often multiple stress conditions. Also, another important layer to be investigated is the context-dependent UPS mediated-regulation acting across distinct developmental stages and under multiple environmental conditions. In this sense, an important challenge is to investigate whether E3 ligases function as key regulatory nodes through which immune signaling and growth-related pathways can be differentially and reversibly modulated, rather than being strictly antagonistic.

Recent advances in proteomics focused on ubiquitination and the identification of degradation motifs, driven by high-throughput approaches such as ubiquitomics and degradomics, have substantially broadened our understanding of the proteins involved in ubiquitination events. These studies move beyond the traditional view of the UPS as a linear pathway dedicated exclusively to protein degradation, instead revealing its role as a highly dynamic regulatory network. With the advent of these technologies, an unexpected breadth of ubiquitination targets has been uncovered, including immune regulators, transcription factors, and cell cycle components, among others. Together, these findings reinforce the notion that the UPS operates as a finely tuned signaling platform rather than a simple on-off control system.

Despite these significant advances over the past decade, functional insights remain disproportionately limited. In particular, the developmental and agronomic consequences of manipulating UPS components through biotechnological strategies are still underexplored. This gap is especially relevant given the growing reliance on genetic and biotechnological approaches to generate pathogen-resistant crops, as well as the widely recognized trade-off between immune activation and plant growth.

As plant biotechnology moves toward increasing precise manipulation of immune pathways, integrating ubiquitination research with developmental and agronomic frameworks will be essential to achieve durable resistance without compromising plant performance.

## Figures and Tables

**Table 1:** Comparison of the major families of E3 ligases used in plant UPS, their structural characteristics, and catalytic mechanisms.

Family	Structure	Ub Transfer Mechanism	Key Function	Example	Articles
HECT (E6-associated protein C-terminus)	HECT domain in the C-terminal region and a variable N-terminal portion (WW, ARM, etc.).	E3 receives ubiquitin at an active cysteine and transfers it to the substrate.	Growth regulation, hormone responses (auxin/ABA) and membrane protein control.	UPL1, UPL3, UPL4 and UPL5.	[27,28,29,30]
RING (Really Interesting New Gene)	RING domain containing Zn^2+^	E3 acts as a platform, allowing direct transfer of E2–Ub to the substrate.	Immunity, stress responses, PCD and receptor modulation.	RHA3A/B, BOI, RARE, RIP1, PIRE, SINA3 CsRGLG4, SlATL2, OsBBI1, OsCUL3a, HUB1 and OsEBR1.	[31,32,33,34,35,36,37,38,39,40,41,42]
U-box	U-box domain lacking Zn^2+^ coordination.	E3 acts as a platform, allowing direct transfer of E2–Ub to the substrate.	PRR regulation, MAPK modulation and negative regulation of immunity.	PUB12/13, PUB25/26 and SPL11.	[43,44,45,46,47]
CRLs (Cullin-RING ligases)	The Complex is formed by Cullin, RBX1 (RING), and F-box adaptor proteins.	E2 associated with RBX1 catalyzes F-box substrate receptor-oriented ubiquitination.	Cell cycle, immunity, hormone signaling (auxin/JA), development and stress responses.	SCF, SCF^EBF^ NPR1, CRL3^NPR3/4^, OsCULLIN3, CRL3^BPM^ and CRL3^LRB^.	[24,48,49,50,51,52,53,54]
APC/C (Anaphase Promoting Complex/ Cyclosome)	Complex with 14–15 subunits, with catalytic subunit APC11 (RING), assisted by APC2 (Cullin-like), and regulatory modules (CCS52 and CDC20).	Direct (E2-substrate) transfer dependent on adaptors (CDC20 and CCS52).	Chromosome separation (securin degradation), cyclin degradation (A, B, and D), regulation of the G2/M transition and mitotic exit.	APC/C^CDC20^ and APC/C^CCS52^ (A1/2).	[55,56,57]

## Data Availability

No new data were created or analyzed in this study. Data sharing is not applicable to this article.
